# Manual small-incision cataract surgery (MSICS): techniques and tips to optimise outcomes

**Published:** 2025-12-02

**Authors:** Mihret Deyesa Kabeta, Lila Raj Puri

**Affiliations:** 1 Senior Ophthalmologist: Ambo General Hospital, Oromia Region, Ambo, Ethiopia.; 2 Medical Advisor, Asia: The Fred Hollows Foundation, London, UK.

**Each step of the MSICS procedure needs to be carried out with great focus and precision in order to ensure a successful outcome.**

Manual small-incision cataract surgery (MSICS) is one of the most common surgical procedures in ophthalmology. It involves several steps, and each step needs great focus to ensure a successful procedure and optimal outcomes. The tips and tricks in this article describe how to optimise MSICS techniques in order to get better uncorrected vision, faster recovery, and fewer complications.

## Anaesthesia

Most cataract operations are performed under local anaesthesia. Peribulbar or retrobulbar anaesthesia is commonly used. Other techniques, like sub-Tenon’s injection, or subconjunctival injection supplemented with intracameral injection, are also used. The overall goal should be to achieve analgesia (absence of pain) and akinesia (absence of eye movement); both are essential for patient comfort and to allow the surgeon to perform delicate procedures safely.

## Preparing the eye for surgery

- Confirm the details of the patient, the intraocular lens (IOL) power, and other information (such as the site and type of procedure) using the preoperative checklist
- Clean the periocular skin and surrounding area using 5% povidone-iodine
- Instil povidone-iodine 5% drops in the conjunctival sac
- Place a sterile eye towel or adhesive drape over the eyelashes and lid margins to isolate them from the surgical field.

## Conjunctival peritomy

- A fornix-based conjunctival flap is usually made at the superior limbus (between 10 and 2 o’clock) However, depending upon the clinical indication, the surgeon’s skill, and the patient’s astigmatism status, a temporal conjunctival peritomy may have to be done
- Tenon’s capsule should be dissected clearly from the incision area
- Apply minimal cautery (preferably wet field) to achieve haemostasis.

## Incision

This is one of the most critical steps of MSICS and determines how easy it will be to deliver the nucleus, as well as the degree of postoperative astigmatism.

The incision size is determined by the type of cataract and size of the nucleus. For example, the extraction of immature cataracts in younger patients may only require a small tunnel, but very big brown nuclei require a larger tunnel.

An incision size of 6–7 mm should be adequate. Longer incisions produce more flattening and lead to against-the-rule astigmatism. The incision should be 1.5–2.0 mm away from the limbus, as posterior incisions decrease against-the-rule astigmatism.

**Figure 1:**
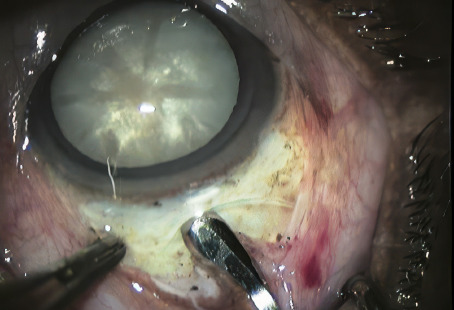
Making a frown-shaped incision

There are different types of incision practiced by eye surgeons, e.g. smile, straight, frown, Blumenthal with side cuts, and chevron V incision. Studies, and our experience, have shown that astigmatism is minimised when using a **frown-shaped incision** (Figure 1) and a **chevron V** incision.

© K HENNIG CC BY-NC-SA 4.0

## Scleral tunnel construction

**Figure 2:**
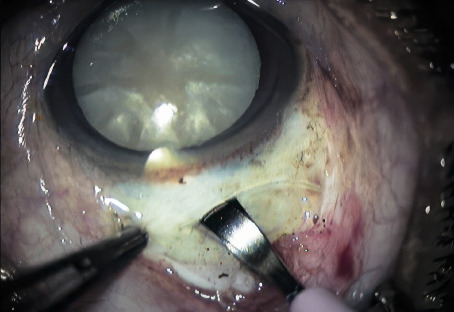
Formation of the scleral tunnel

- Construct the scleral tunnel using a sharp crescent blade (Figure 2). It should extend approximately 1.0–1.5 mm into the cornea.
- Carry out the dissection towards the limbus on both sides to create a funnel-shaped ‘pocket’, maintaining a uniform depth of approximately 50% of scleral thickness. This is because a too-shallow incision can cause buttonholing and a too-deep incision can cause premature entry. Be careful not to cut the limbus on either side.
- Create side pockets at the ends to enlarge the internal opening, especially when delivering a large nucleus.
- Keep the tunnel ‘shelved’, with the external opening smaller than the internal, to maintain the valve effect.

© K HENNIG CC BY-NC-SA 4.0

## Entry into anterior chamber

- Use a 3.2 mm keratome with a sharp tip to enter the anterior chamber, as a blunt tip may cause detachment of Descemet’s membrane. Check the tip before using it.
- Enter through the internal corneal lip of the tunnel, and follow the corneal plane up to 1.5–2.0 mm before changing direction towards the anterior chamber. If the anterior chamber is entered before the corneal lip is reached, then the valve effect is lost and a suture will be required at the end of the surgery. The internal opening is usually 8–10 mm larger than the external scleral incision.
- Take care not to dissect too far towards the anterior during scleral tunnel creation and internal incision extension, as it can lead to injuries to the iris or ciliary body, causing them to bleed. During and after preparing the tunnel, maintain pressure in the anterior chamber using viscoelastic.

## Side-port incision

Create a separate small side-port (paracentesis) using a keratome or 15-degree knife. This is needed for the introduction of the second instrument for irrigation aspiration, IOL dialling, injection of viscoelastics, capsulotomy, removal of subincisional cortex, intracameral injection, and anterior chamber reformation at the end of surgery. Again, ensure the tip of the keratome or 15-degree knife is sharp and not bent; this will help to avoid detachment of Descemet’s membrane. The ideal size of the side-port incision is around 1.5 mm. The size is important, as a too-small incision creates difficulty during instrument manipulation and capsulotomy, and too-large incision may lead to instability of the anterior chamber and problems during anterior chamber formation at the end of surgery.

## Capsulotomy

Improper capsulotomy may lead to uncontrolled capsular tear extension, posterior capsule rupture, vitreous loss, IOL decentration, IOL drop, and even nucleus drop during hydroprocedure.

**Figure 3:**
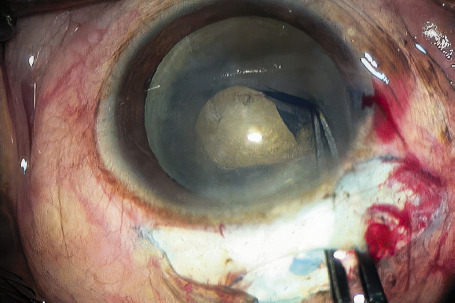
Continuous curvilinear capsulorhexis

The best capsular opening is a **continuous curvilinear capsulorhexis (CCC)**, as it will guarantee long-term, ‘in the bag’ IOL centration (Figure 3). However, in mature and hypermature cataracts, and in intumescent cataracts, the **envelope technique** is preferable due to the safety it provides to the corneal endothelium during nucleus delivery. Other techniques may be needed, depending upon the pupil size, nucleus size, the experience of the surgeon, and the availability of instruments.

© K HENNIG CC BY-NC-SA 4.0

It is always advisable for less experienced surgeons to use tryphan blue to stain the capsule before carrying out the capsulotomy, as this makes it easier to see the shape of the capsule and avoid extension into the posterior capsule.

The size of the capsulotomy should be 5–6 mm. If the capsulorrhexis is small and an attempt is made to deliver a large nucleus through the small opening, zonular dialysis and zonular rupture is likely.

Maintaining the anterior chamber throughout the procedure with liberal use of viscoelastic is crucial for better outcomes.

## Hydroprocedure

Good hydrodissection is very important to achieve nucleus mobility and avoid prolapse of the anterior chamber.

**Figure 4:**
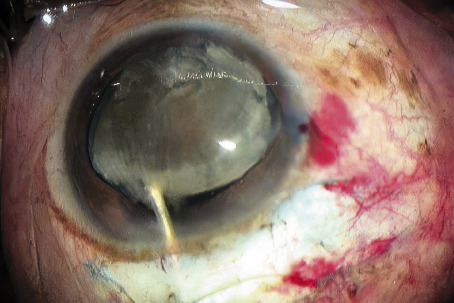
Mobilising the nucleus before lifting it into the anterior chamber

- Form the anterior chamber with cohesive viscoelastics but leave a small egress path (don't trap fluid)
- Position the cannula between the capsule and nucleus
- Give small, pulsed injections of fluid and gentle decompression. It is advisable to do multi-quadrant hydrodissection
- In posterior polar, traumatic, mature, and hypermature cataracts, perform hydrodissection gently and gradually to avoid complications like zonular dialysis, capsule tear, capsulotomy extension, and nucleus drop
- At the end of the procedure, rotate the nucleus gently to confirm mobility (Figure 4).

## Nucleus delivery

The basic principle is to take out the nucleus from the capsular bag and main wound without damaging the corneal endothelium and capsule.

**Figure 5:**
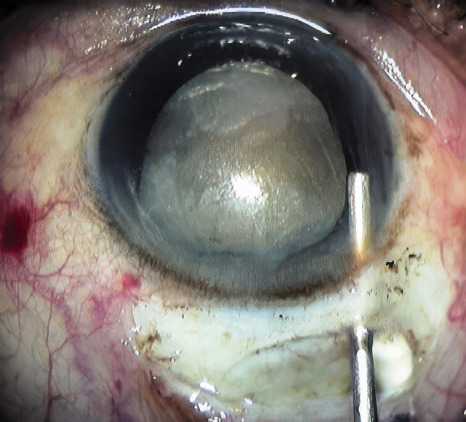
Checking the size of the tunnel

- Check the size of the tunnel (Figure 5). If the incision is too small for nucleus delivery, slightly enlarge the tunnel to prevent undue stress on the zonules, rotation or flipping of the nucleus, and corneal endothelial touch.
- Once a pole or nucleus is out, visco-coat the endothelium, then complete the nucleus delivery via your preferred method (such as visco expression, irrigating Vectis, fishhook technique, Simcoe cannula, or hydro expression).
- Use viscoelastic to form the anterior chamber, push the iris, and prevent the nucleus from touching the corneal endothelium.

© K HENNIG CC BY-NC-SA 4.0

“It is always advisable for less experienced surgeons to use tryphan blue to stain the capsule before carrying out the capsulotomy.”

© K HENNIG CC BY-NC-SA 4.0

## Cortex removal

Most of the lens cortex can be removed with a Simcoe cannula through the tunnel and side-port incision. Strip the cortex circumferentially, pulling centrally to avoid capsular stress. A sub-incisiona cortex can be safely aspirated through a side port. If stripping of Descemet’s membrane occurs while cleaning the cortex, take great care not to tear it off. If this happens, inject air into the chamber at the end of the operation to push Descemet’s membrane against the cornea.

While clearing the cortex with a Simcoe cannula, posterior capsule rupture and vitreous loss may occur. This can be avoided by carefully watching the posterior capsule.

To reduce the risk of a postoperative increase in intraocular pressure, thoroughly remove all viscoelastic. If there is vitreous in the anterior chamber, cortex removal should be done after anterior vitrectomy and vitreous removal. The capsular bag must be free of cortex at the end of the procedure in order to minimise postoperative inflammation, prevent posterior capsular opacification, and optimise visual outcomes.

“MSICS remains a high-quality, scalable, and cost-effective technique for cataract surgery. Visual outcomes depend less on technology and more on meticulous technique.”

## IOL implantation

Before implanting the IOL in the capsular bag, check the IOL type and power and ensure that the sclerocorneal tunnel is adequate. The capsule should be intact or there should be adequate capsular support to place the IOL (in the sulcus in case of posterior capsule rupture). The capsular bag should be filled with viscoelastic to expand it and to protect the endothelium.

Enlarge the tunnel slightly if IOL implantation is difficult, to avoid damaging the haptics or bag.

### Tips:

- Always re-inject viscoelastic before IOL insertion to avoid capsular trauma.
- Never push the IOL blindly – visualise both haptics entering the bag.
- If there is resistance during insertion, enlarge the tunnel slightly rather than forcing the lens.
- After IOL placement, check for pupil roundness, optic centration, and wound integrity.

## Wound closure

After clearing the viscoelastic from the anterior chamber and from behind the IOL (which requires greater skill), close the wound by hydrating via the side port. Use the peritomised conjunctiva to cover the scleral incision; ensure it will remain in place. Exposure of the incision may increase the risk of infection as well as patient discomfort.

## Suturing

Proper tunnel construction is the best closure. However, do not hesitate to place a suture if needed; this is better than risking hypotony, wound leak, or infection. Suturing is required if:

- The tunnel is too short, ragged, or extended
- There are intraoperative complications (e.g. scleral thinning or premature entry)
- There is an unstable wound, especially in patients who have high myopia or have already lost sight in one eye
- There is a persistent and significant leak.

## Final steps

The final steps of MSICS surgery are:

- Instil intracameral antibiotic
- Form the anterior chamber one last time using balanced salt solution
- Apply subconjunctival steroid and antibiotic
- Pad the eye.

### Tips:

- Always check for a watertight seal with Seidel’s test before ending the operation.

## Conclusion

MSICS remains a high-quality, scalable, and cost-effective technique for cataract surgery. Visual outcomes depend less on technology and more on meticulous technique.

Optimisation lies in small refinements: creating the tunnel correctly, a well-centred capsulorrhexis, gentle nucleus delivery, complete cortex removal, stable IOL placement, and a watertight wound. With training, vigilance, and outcome monitoring, MSICS can deliver outcomes comparable to phacoemulsification, while serving the greatest number of people in need.

### Further reading

Community Eye Health Journal Volume 21 Issue 65 Cataract complications. bit.ly/4nEiapw

